# Battle of epigenetic proportions: comparing Illumina’s EPIC methylation microarrays and TruSeq targeted bisulfite sequencing

**DOI:** 10.1080/15592294.2019.1656159

**Published:** 2019-09-05

**Authors:** Jonathan A. Heiss, Kasey J. Brennan, Andrea A. Baccarelli, Martha M. Téllez-Rojo, Guadalupe Estrada-Gutiérrez, Robert O. Wright, Allan C. Just

**Affiliations:** a Department of Environmental Medicine and Public Health, Icahn School of Medicine at Mount Sinai, New York, NY, USA; b Department of Environmental Health Sciences, Mailman School of Public Health, Columbia University Medical Center, New York, NY, USA; c Center for Nutrition and Health Research, National Institute of Public Health, Cuernavaca, Mexico; d National Institute of Perinatology, Mexico City, Mexico

**Keywords:** DNA methylation, EWAS, microarray, bisulphite sequencing, comparison of experimental platforms

## Abstract

DNA methylation microarrays have been the platform of choice for epigenome-wide association studies in epidemiology, but declining costs have rendered targeted bisulphite sequencing a feasible alternative. Nonetheless, the literature for researchers seeking guidance on which platform to choose is sparse. To fill this gap, we conducted a comparison study in which we processed cord blood samples from four newborns in duplicates using both the Illumina HumanMethylationEPIC BeadChip and the Illumina TruSeq Methyl Capture EPIC Kit, and evaluated both platforms in regard to coverage, reproducibility, and identification of differential methylation. We conclude that with current analytic goals microarrays still outperform bisulphite sequencing for precise quantification of DNA methylation.

## Introduction

Epigenetics is believed to play a key role in many associations observed between environmental exposures and health and developmental outcomes, e.g. lead exposure and low birth weight [1] or maternal smoking and childhood asthma [2]. Even though the impact of toxic environmental exposures on populations can be quite substantial due to their high prevalence, on an individual level their effect sizes are often small in part because health outcomes are multi-factorial. This is reflected on the genetic/epigenetic level where polygenes and pleiotropy are the norm and clear-cut links between geno/epitypes and phenotypes are rare, complicating discovery and validation of exposure-related epigenetic changes. It is, therefore, crucial to have the best laboratory tools available. Discovery is usually carried out in the form of epigenome-wide association studies: potentially millions of CpG sites are scanned in a hypothesis-free manner for their association with an outcome or exposure of interest. While DNA microarrays have been the platform of choice, technical advancements and declining costs have rendered targeted bisulphite sequencing a feasible alternative, raising the question of whether investigators should consider switching to targeted bisulphite sequencing for new projects. Both offer single-site resolution of DNA methylation levels and genome-wide coverage but differ in regard to precision and density. Microarrays and targeted bisulphite sequencing have been compared and their respective (dis)advantages have been discussed previously [3,4], these reports, however, do not include benchmarks addressing the typical scenario encountered in environmental epidemiology, which is usually characterized by rather large sample sizes but small effect sizes, and therefore provide little guidance for researchers in this field planning new studies. The aim of this project was to fill this gap by comparing both platforms as directly as possible.

## Methods

DNA methylation levels were measured in cord blood samples from four newborns, two boys and two girls. Samples were run in duplicate (one index sample and one technical replicate) on microarrays as well as using targeted bisulphite sequencing, amounting to a total of 16 experiments. Both platforms were evaluated in regard to reproducibility between duplicates and well as the ability to rediscover CpG sites differentially methylated between sexes.

### Study cohort

Cord blood samples were collected in the context of a larger birth cohort study named PROGRESS. In total, 948 pregnant women, recruited before the second trimester and residing in Mexico City, were enrolled between July 2007 and February 2011 and gave birth to a live infant. They were followed throughout pregnancy, delivery and beyond, with data and sample collection from both mothers and newborns at several time points. The study protocol was approved by the institutional review boards of the Icahn School of Medicine at Mount Sinai, Harvard School of Public Health and the Mexican National Institute of Public Health (INSP) and all participants provided written informed consent. Methylation microarray data of whole blood DNA extracted from umbilical cord blood samples were available for 449 newborns.

### Microarray data

Samples from PROGRESS were assayed on the Illumina MethylationEPIC BeadChip (**EPIC**, Illumina Inc., San Diego, CA). EPIC experiments were run at the University of Illinois following bisulphite conversion at Northwestern University according to the manufacturer’s protocol: DNA input is bisulphite-converted, amplified, fragmented, subsequently dropped on the array where the targeted DNA molecules hybridize to the probe sequences, extended with tagged nucleotides, and finally scanned to record fluorescence intensities. Generated output is saved in .idat files, the starting point for this analysis. Samples passed comprehensive quality control [5] and undetected probes were filtered out (estimating background noise using non-specific fluorescence instead of negative control probes) [6] and fluorescence intensities were corrected for dye bias using RELIC [7]. Fluorescence intensities were converted into methylation levels ***β_A_***[8]. No normalization was performed. Four subjects were chosen randomly out of the pool of samples that passed, had a sufficient amount of DNA, and had been measured in duplicates, each with index and technical replicate placed on different 96-well plates and different rows on the 8-well chips.

### Bisulphite sequencing

Samples were sequenced at the Epigenomics Core Facility at Weill Cornell Medicine using the Illumina TruSeq Methyl Capture EPIC Kit (**TruSeq**) and preprocessed as described previously [9]. TruSeq uses targeted enrichment in which baits hybridize to regions of interest for subsequent amplification. Samples were run on an Illumina HiSeq 2500 with 100 bp paired-end sequencing on two lanes with a total yield of 66 Giga bases. Preprocessing included quality control using *FastQC*, adapter trimming using *cutadapt*, and read alignment and methylation calling using *Bismark* with *Bowtie2 (command-line arguments: -q – score-min L,0,-0.2 – ignore-quals – no-mixed – no-discordant – dovetail – maxins 500)*. No de-duplication was performed as recommended for targeted sequencing. The starting point for this analysis were methylated and total counts of reads overlapping queried CpG sites. In the context of sequencing the term coverage can possess several meanings. To avoid confusion we use the term read depth in order to refer to the total number of sequenced reads that map to/overlap a CpG site. We use the term coverage exclusively to convey whether a CpG site is targeted/queried at all by either platform.

Whereas for EPIC only one preprocessing pipeline and therefore only one set of methylation level estimates was produced, three sets of estimates were evaluated for TruSeq. But beforehand a list of CpG sites common to EPIC and TruSeq was compiled. We will refer to this set of CpG sites as the reference loci. First, raw methylation levels ***β_R_*** were estimated as the ratio of methylated to total counts for each particular CpG site (hence the ‘*R*’ in ***β_R_***). Second, blocks were defined by assigning CpG sites to the nearest reference locus. Only sites within 250bp in either direction of a reference locus were considered, capping block size at 501bp. This window size was chosen to allow sufficient numbers of CpG sites per block to take advantage of pooling while only grouping together CpG sites that are close enough to be highly correlated and likely part of the same functional unit. For each block, methylated and total counts were tallied and their ratio ***β_C_*** used as an estimate representative of the collapsed methylation proportion in the entire block (hence the ‘*C*’ in ***β_C_***). The genomic position of the reference locus was used to match blocks to EPIC probes. Third, smoothed estimates ***β_S_*** for each CpG site were produced by applying the *BSmooth* function from the frequently cited *bsseq* Bioconductor package with default parameters (hence the ‘*S*’ in ***β_S_***) [10]. The BSmooth function performs locally weighted regressions taking the read depth into account. By its design, it enables the imputation of missing values.

### Reproducibility

Only CpG sites common to EPIC and TruSeq were considered in this step and CpG sites mapping to the X and Y chromosomes were dropped as well. Reproducibility was measured by the Pearson correlation coefficient of methylation levels between index samples and technical replicates across all probes, before and after centring the data by subtracting CpG-specific means [11]: in the pursuit of differential methylation only inter-sample variation is relevant. Due to the bimodal distribution of methylation levels and the fact that many CpG sites exhibit little biological variation, intra-sample variation (i.e. between different CpGs in the same sample) greatly outpaces inter-sample variation (i.e. between different samples for the same CpG). In this situation correlation of non-centred data conveys little information about reproducibility. For example, correlation coefficients between pairs of EPIC arrays without centring usually range above 0.98 even for unrelated samples. Data were not standardized as measurements are already on the same scale (from 0 to 100% methylation).

### Discovery of differential methylation

All EPIC data of cord blood samples from PROGRESS save the four subjects with TruSeq data (resulting in a sample size of 445) were used to compile a list of CpG sites differentially methylated between boys and girls *(positive markers)* and a second list consisting of not-differentially methylated sites *(negative markers)*. Only autosomal CpG sites common to EPIC and TruSeq were considered and potentially cross-hybridizing probes, as judged by sequence homology [12], were excluded. ***β_A_*** were regressed (linearly) on sex, batch (i.e. the 96-well plates on which samples were allocated), and leukocyte composition. Proportions of seven cell types (granulocytes, monocytes, natural killer cells, CD19 + B-lymphocytes, CD8 + T-cells, CD4 + T-cells, nucleated red blood cells) were estimated using the Houseman algorithm [13] based on two reference datasets of purified cord blood cells [14,15]. CpG sites with p-values below 0.05 after Bonferroni correction were deemed positive, CpG sites with p-values above 0.1 before Bonferroni correction were deemed negative. Positive markers were further restricted to absolute effect sizes capped at 0.05 as we wanted to focus on small effect sizes. The list of positive and negative markers is referred to as *ground truth* for this study.

Association between methylation levels in cord blood and sex was again assessed in the subset consisting of eight (the four subjects with duplicate measurements not used to define the ground truth) EPIC samples and eight TruSeq samples, respectively. Five analytical approaches were employed depending on which set of estimates was used. Conditional on what yielded better performance, either linear or logistic regression was used.

- For ***β_A_*** logistic regression models with sex as the only independent variable (aside from the intercept)
- For ***β_C_*** logistic regression models with sex as the only independent variable and observations weighted by read depth.
- For ***β_R_*** logistic regression models with sex as the only independent variable and observations weighted by read depth. Only reference loci were considered.
- Again using ***β_R_*** and logistic regression models with weighted observations, but with separate models for every CpG site of a block, not just the reference loci. Subsequently p-values were summarized by block into a single p-value using Fisher’s method.
- For ***β_S_*** linear regression models and subsequently summarizing of p-values by block with Fisher’s method. No weighting of observations was done as read depth is already taken into account during the smoothing step.

These approaches differ whether information is pooled across CpG sites or not, and whether methylation levels or p-values are pooled. While not exhaustive, they represent the various basic strategies of regional methylation analysis, with the use of ***β_S_*** representing a hybrid approach. Using the corresponding p-values as scores, the ability to discriminate between positive and negative markers, as determined by the population-level EWAS, was measured with the *c*-statistic.

## Results

### Coverage

**Figure 1.**
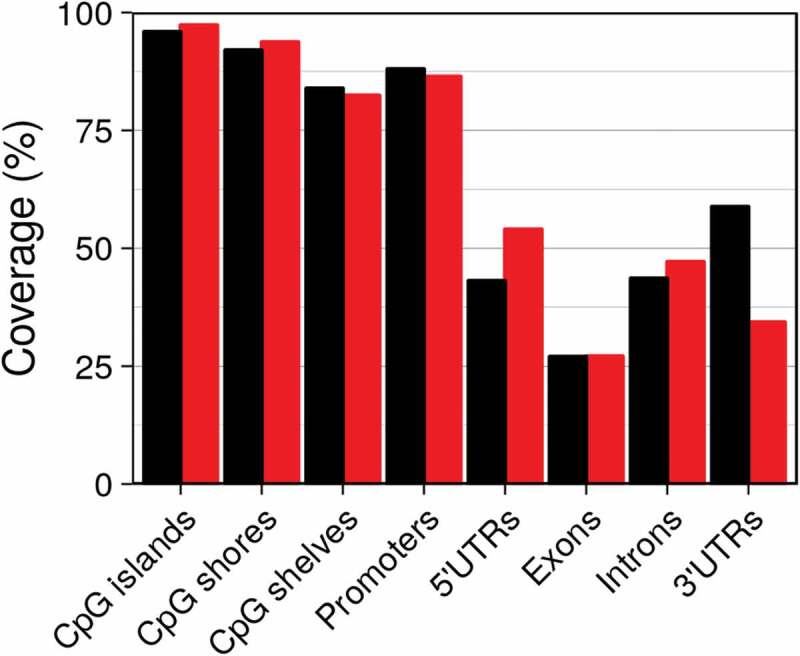
Coverage of epigenetic and genetic elements for EPIC (black) and TruSeq (red).

Not counting those targeting non-CpG sites, there were 862,927 probes on the EPIC, amounting to 3% of the roughly 28.2 million CpG sites in the human genome (version hg19). TruSeq enriches for 437,792 preselected regions encompassing 3,358,636 or 12% of all CpGs. TruSeq builds upon the content of the EPIC chip, resulting in a high overlap between both platforms. When we overlapped CpG sites/probes with annotation tracks of epigenetic elements (with categories of CpG island, shore, shelf) and genetic elements (3ʹ UTR, 5ʹ UTR, promoter, exon, intron), we found that despite querying roughly four times the number of CpG sites compared to EPIC, the percentage of (epi)genetic elements covered (overlap with at least one probe/CpG site) by TruSeq was similar (see Figure 1). Both platforms cover the vast majority of CpG islands, shores and shelves. Coverage of the various genetic elements was less complete with the highest coverage for promoters and the least for exons.

As a result of the random assembly manufacturing process of the EPIC chip, some probes might not actually be present and have to be masked in the data. Furthermore, a small fraction of probes are usually masked as they show very low fluorescence intensities, an indication that the targeted CpG site has not been sufficiently amplified or might differ from the reference genome (either because the CpG site itself is mutated/deleted or because nearby changes prevent hybridization to the 50-mer probes) so that the estimated methylation levels represent spurious values. Similarly, some of the CpG sites targeted by TruSeq have no reads mapped to them. In our dataset these instances were rare, affecting only 1.0% and 0.3% of EPIC and TruSeq data, respectively.

### Read depth

Median read depth at CpG sites targeted by TruSeq was 51 with the following distribution: 0.3% at read depth 0 (missing), 2.5% at 1–10, 8.5% at 11–20, 12.9% at 21–30, 13.6% at 31–40, 12.2% at 41–50, and 49.9% at 51–300, and 0.1% at read depths above 300. Reads mapped outside the targeted regions were dropped before continuing with the analysis, even when corresponding EPIC probes were available, as median read depth was 0. CpG sites with read depths below 11 or above 300 were filtered out before evaluating reproducibility and ability to detect differential methylation.

### Concordance

**Figure 2.**
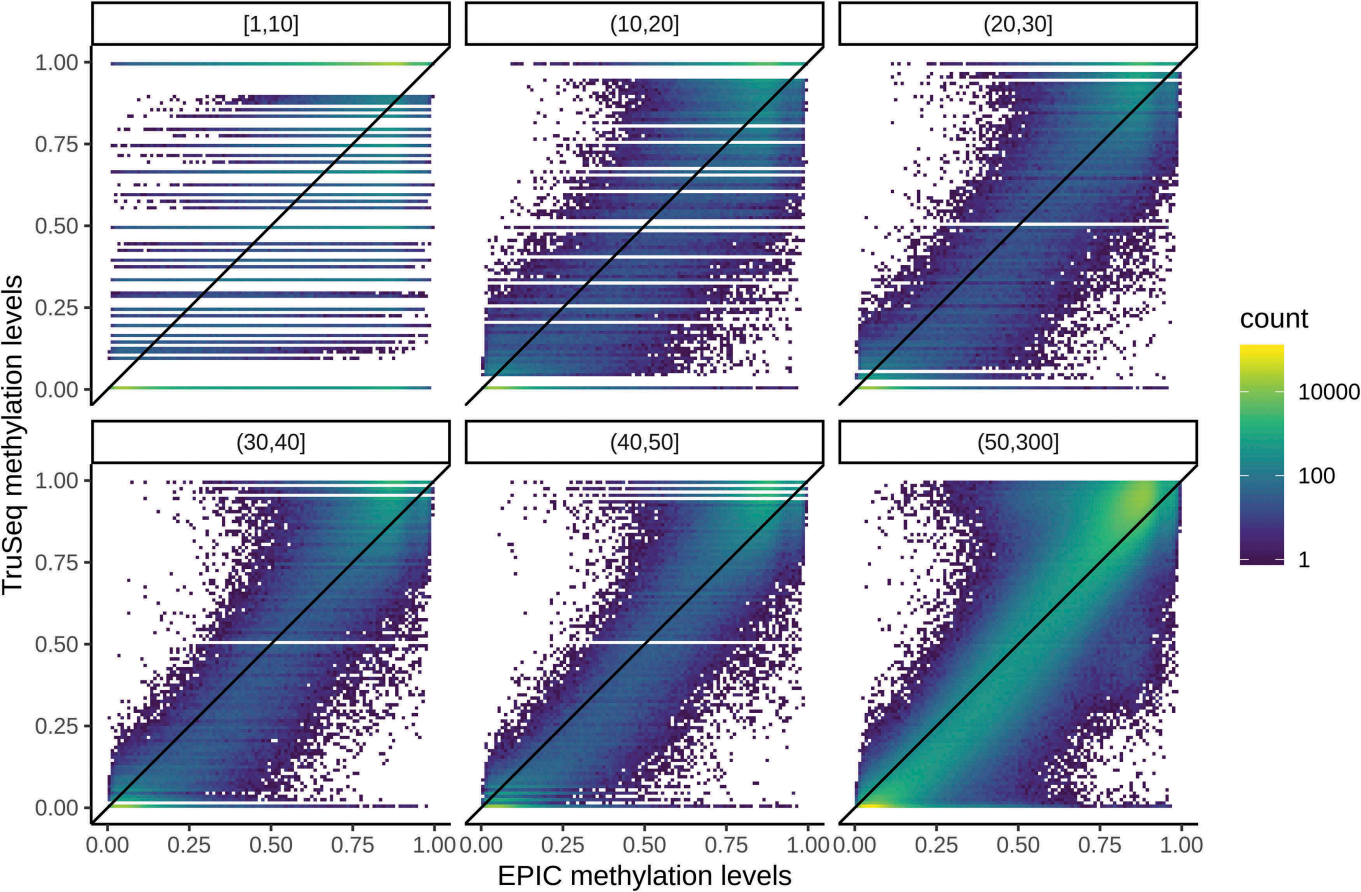
Concordance of methylation level estimates between EPIC and TruSeq samples stratified by read depth.

Figure 2 shows the concordance of estimated methylation levels between EPIC and TruSeq samples stratified by read depth across all eight pairs. As expected, concordance (as assessed by the correlation between 5,220,451 paired measurements) improves with increasing read depth, ranging from 0.835 (1–10 mapped reads) to 0.980 (51–300 mapped reads). The discrete nature of bisulphite sequencing data is especially apparent for lower read depths. Even though most CpG sites are completely (un)methylated, the peaks of the bimodal distribution of EPIC methylation levels are shifted inwards.

### Reproducibility

In the case of TruSeq, precision is primarily a function of read depth (see Figure 2). After filtering out allosomal loci and TruSeq data with low or excess reads (<11 or >300) there were a total of 570,494 remaining CpG sites in common between platforms. Correlation coefficients between index samples and technical replicates using non-centred data were 0.997 for ***β_A_*** and 0.986, 0.991 and 0.993 for ***β_R_***,***β_C_*** and ***β_S_***, respectively. After centring the data these coefficients changed to 0.493 for ***β_A_*** and 0.178, 0.259 and 0.250 for ***β_R_***, ***β_C_*** and ***β_S_***, respectively. The necessity to centre the data becomes even more clear by looking at unrelated samples (which should not be as closely correlated): matching each index sample with the wrong technical replicate of the same sex, correlation coefficients were 0.993 for ***β_A_*** and 0.980 for ***β_R_*** when using the non-centred data, and −0.163 for ***β_A_*** and −0.149 for ***β_R_*** when using the centred data.

Further restricting the TruSeq centred data to sites with a read depth greater than 50 resulted in correlation coefficients of 0.273 for ***β*_R_**. To achieve a correlation coefficient for ***β*_R_** as high as for ***β*_A_** it was necessary to restrict the data to those with a read-depth above 130 (~10% of data, median read depth of 146).

### Differential methylation

After dropping probes that are potentially cross-reactive, methylation levels ***β_A_*** of reference loci were tested for their association with sex in the remaining n = 445 cord blood samples from the PROGRESS cohort. A total of 10,495 probes were differentially methylated between boys and girls at a family-wise error rate of 0.05, while 404,106 probes had unadjusted p-values above 0.1. We further restricted the set of positive markers to 9,653 with absolute effect sizes below 0.05 on the *β*-scale. The ability to discriminate between the two lists of positive and negative markers was evaluated in the EPIC and TruSeq datasets, each consisting of eight samples (four subjects run in duplicates). The results are presented in Table 1. The highest c-statistic was achieved for EPIC (I), the lowest for TruSeq with smoothed methylation estimates (V). Pooling p-values or methylation levels modestly improved discrimination (IV vs. III and II vs. III).

**Table 1.** Results of differential methylation benchmark.

Approach #	Platform	Estimates	Pooling of methylation levels	Pooling of p-values	Model	c-statistic
I	EPIC	***β_A_***	No	No	Logistic regression	0.761
II	TruSeq	***β_C_***	Yes	No	Weighted logistic regression	0.663
III	TruSeq	***β_R_***	No	No (reference loci only)	Weighted logistic regression	0.647
IV	TruSeq	***β_R_***	No	Yes	Weighted logistic regression	0.675
V	TruSeq	***β_S_***	Yes	Yes	Linear regression	0.586

## Discussion

Out of the rich selection of platforms for quantification of DNA methylation, EPIC and TruSeq share performance characteristics and costs that render them suitable for epigenome-wide association analyses. But both platforms come with their own challenges regarding preprocessing, batch effects, and statistical analysis. No consensus on best practices has emerged yet and an ever-growing selection of available software packages only makes the decision more difficult. Many open questions remain, most fundamentally how to segment or group CpG sites? (An overview of existing methods can be found in Chen et al., 2016 [16].) While often highly correlated, sudden changes in methylation levels between proximal CpG sites can be observed. Despite these unresolved issues, investigators planning new studies still have to decide on platforms and analytic strategies in the meantime.

We wanted to answer the question of which platform, EPIC or TruSeq, provides the most information in the context of epigenome-wide association studies of environmental exposures, which are usually characterized by small effect sizes. While DNA methylation microarrays query only a small fraction of all CpG sites in the human genome, they offer better precision, whereas bisulphite sequencing techniques can query more CpG sites – even the entire genome – but usually do so at low coverage and consequently low precision due to cost constraints. In terms of genome-wide coverage both platforms perform very similarly. To facilitate a more useful comparison, we restricted our analysis to common CpG sites/regions and rephrased the question more specifically as to whether the lower precision of bisulphite sequencing data can be compensated by pooling information from nearby CpG sites and whether such a strategy might even improve detection of differential methylation? Again, while the answer might depend on the magnitude of the sought-after epigenetic differences, we were most interested in small-magnitude effect sizes.

We chose to apply rather lightweight preprocessing and simple analytical approaches. In the case of EPIC, this included dye bias correction (a within-array method which nevertheless improves between-array reproducibility [7]) and filtering by detection p-values, but no normalization or regional analyses. In the case of TruSeq, this included using the default preprocessing for bisulphite sequencing data as performed at the Epigenomics Core Facility at Weill Cornell Medicine [9], and simple analytical approaches of pooling information from nearby CpG sites. While not exhaustive, the selection of analytical approaches evaluated here covers all of the basic strategies.

Both platforms performed similarly in regards to coverage of genetic and epigenetic elements, and the fraction of missing data, i.e., the number of targeted CpG sites that were ultimately not observed was low, an important feature when the same CpG sites are to be compared across hundreds of samples.

Comparing reproducibility of single-site methylation level estimates, we find that EPIC clearly outperforms TruSeq with a correlation coefficient after mean-centering of 0.493 compared to 0.178. While pooling methylation levels across nearby CpG sites did improve the correlation to 0.259, this is still substantially worse than for the EPIC data. It is crucial to note that correlation coefficients were evaluated using centred data: previous comparisons of bisulphite sequencing and microarray methylomic assays reported Pearson correlations of non-centred data that are misleadingly high.

Consistent with these findings, the best performance in the differential methylation benchmark was achieved using EPIC data, yielding a c-statistic of 0.761. For TruSeq, pooling p-values or reads from nearby CpG sites gave only slightly better results (0.675 and 0.663) than just using the reference loci (0.647). Interestingly, the methylation level estimates produced by BSmooth, even though scoring better in regard to reproducibility, yielded the worst c-statistic (0.586).

There are strengths and important limitations to our analysis. We relied on a large dataset in order to compile the ground truth for the differential methylation benchmark. Another common benchmark strategy, in case no ground truth is available, is to split a dataset, run the same analytical approach on each half independently, and subsequently check results for consistency. The so-called correspondence-at-the-top metric represents such a benchmark: CpG sites are ranked by increasing likelihood of differential methylation and the overlap between the top *n* hits from each half is counted. We refrained from such a strategy, as for TruSeq p-values do not only depend on the magnitude of the effect size but also read depth. The non-uniform non-random distribution of read depth across genomic regions has the potential to skew the ranking (especially considering the small sample size) and thus give a false impression of the ability to identify actual differential methylation. Ground truth based benchmarks do not suffer from such problems. However, ground truth was here determined also using the EPIC platform and some of the classifications of positive and negative markers may not be valid for TruSeq, for example, because of cross-reactivity (even though we tried to mitigate this issue by dropping potentially problematic probes beforehand) and may, therefore, bias this benchmark in favor of EPIC.

When grouping CpG sites queried by TruSeq into blocks, they were matched to the nearest EPIC probe within 250bp if available. This segmentation ignores the underlying methylation levels and may break up functional units even when established analytical approaches would group them together and may result in a loss of statistical power. In case such an unfavorable scenario would stem from closely placed EPIC probes, however, pooling information from those probes would likely have resulted in improved statistical power as well. In other words, if an optimal analytical approach for TruSeq data would group together two blocks, so would an optimal analytical approach likely group together the corresponding EPIC probes. The segmentation based on genomic proximity would therefore not give either platform a competitive edge. However, there may be better ways to define functionally-related proximal sites which could, in general, improve results from smoothing or pooling approaches [17].

Most limiting is the small sample size of just four unique subjects, which does not resemble the situation of a typical EWAS which often includes hundreds of samples. This limitation led us to treat duplicates as independent observations in the differential methylation benchmark, which can lead to an incorrect distribution of p-values. It would do so however for all analytical approaches considered here. Furthermore, the c-statistic assesses the discriminative power, i.e. the relative ‒ not absolute ‒ ranking of positive compared to negative markers. The small sample size is also the reason why we do not report confidence intervals, as such would have required a resampling scheme to accurately reflect variability.

In addition, we would like to make a few comments regarding each platform not addressed in our analysis. While regional analytical approaches exist for EPIC (and indeed have been applied where probe density allows [18,19]), methylation levels are not comparable between probes. Sequence homology between probes leads to off-target binding/cross-hybridization which explains the reduced dynamic range of methylation levels compared to TruSeq as seen in Figure 2. The magnitude of off-target binding and thus the intensity of background noise depends on the probe sequence and therefore varies even for probes that are near each other in genomic coordinates. Another source of noise is cross-talk between the green and red fluorophores used to distinguish the different nucleotides incorporated in the single-base extension step [8]. This issue mainly affects probes of Infinium Type II design [6] which make up 84% of the EPIC array, resulting in an even narrower dynamic range of Infinium Type II probes compared to Infinium Type I probes. Regional approaches based on pooling single-site p-values may still be valid for EPIC, though. In comparison, TruSeq offers more accurate methylation levels, as reads that do not map uniquely are discarded. Compared with whole-genome bisulphite sequencing, the TruSeq targeted enrichment approach can offer a considerably greater read depth with excellent coverage of the Illumina targeted regions. Yet even with this read depth (median of 51 reads per targeted CpG), the precision of bisulphite sequencing data cannot match that from microarrays which have many thousands of oligo binding sites per bead. Last, it should be mentioned that using the 450K/EPIC microarrays ensures comparability to the vast body of literature and reference datasets using the same platform.

## Conclusions

In the context of epigenome-wide association studies of environmental exposures, methylation microarrays are still the laboratory platform of choice. Pooling information from nearby CpG sites cannot compensate for the lower precision of single-site methylation levels, at least not at average read depth around 50.
